# Early Menopause May Associate With a Higher Risk of CKD and All-Cause Mortality in Postmenopausal Women: An Analysis of NHANES, 1999–2014

**DOI:** 10.3389/fmed.2022.823835

**Published:** 2022-03-18

**Authors:** Duo Qian, Zu-feng Wang, Yi-chun Cheng, Ran Luo, Shu-Wang Ge, Gang Xu

**Affiliations:** Division of Internal Medicine, Department of Nephrology, Tongji Hospital, Tongji Medical College, Huazhong University of Science and Technology, Wuhan, China

**Keywords:** menopause, women, chronic kidney disease, oophorectomy, mortality

## Abstract

**Background:**

Chronic kidney disease (CKD) in women is often accompanied by hormone disorders such as sex hormones, and most women with CKD are in the post-menopausal age group. Due to the close relationship between menopause and sex hormones, we aimed to explore the association between early menopause and CKD in post-menopausal women, and the influence of early menopause on longevity in the CKD population.

**Methods:**

Information regarding 4,945 post-menopausal women was extracted from the database of the National Health and Nutrition Examination Survey (NHANES) 1999–2014, and then divided into 4 groups according to the type of menopause (natural or surgical) and early menopause (menopause at age <45) or not. The association between early menopause and CKD prevalence was examined using multivariable logistic regression, while we used multivariable Cox proportional hazards models to investigate the possible relationship between early menopause and all-cause mortality in CKD and non-CKD populations. The differences in the levels of sex hormones between women with and without CKD were also explored.

**Results:**

Compared with women with natural menopause at age ≥45, women experiencing early natural menopause had a higher risk of CKD [OR = 1.26 (1.01–1.56)]. Similarly, as compared to women with surgical menopause at age ≥ 45, women in the early surgical menopause group were more likely to have CKD [OR = 1.38 (1.05–1.81)]. In addition, early surgical menopause was associated with higher mortality in the non-CKD group [HR = 1.62 (1.06–2.49)], but not in the CKD group. Women with CKD had a higher level of luteinizing hormone and follicle-stimulating hormone, combined with a lower level of testosterone and estradiol than the non-CKD women.

**Conclusion:**

Both early natural and surgical menopause were associated with a higher risk of CKD. Early surgical menopause was a hazard factor for survival in the non-CKD group, but not in the CKD group. Further research is required to understand the mechanisms.

## Introduction

Chronic kidney disease (CKD), mainly manifested by decreased GFR and proteinuria (1), is a major public health problem. In the United States, 14.5% of the population meets the criteria for CKD (2). In women with CKD, hormone disorders such as sex hormones disorders are highly prevalent, which may lead to some gynecological problems, for example, menorrhagia and early menopause (3, 4). Moreover, compared with the mean age of 48.8 years at menopause in the general population (5), women with end-stage kidney disease (ESKD) have earlier menopause at a mean age of 45.9 years (6).

Previous studies have shown that early menopause, both natural and surgical, is associated with many diseases, including cardiovascular disease, diabetes, and osteoporosis (7–11). Considering the protective role of estrogens on the cardiovascular system, bone density, and insulin resistance, the shorter lifetime exposure to endogenous estrogens may be the main reason (7, 9). As a consequence, early menopause may influence lifespan (12, 13). Estrogen also appears to be renoprotective in women (14) and it is possible that early menopause is associated with a higher risk of CKD and may affect survival in CKD patients as a consequence of endocrine disturbances. However, the association of early menopause throughout the stages of CKD is inconclusive, and the information about early menopause and mortality in CKD patients is lacking.

We hypothesized that early menopause might be a risk factor of CKD and associated with higher mortality among women with CKD. We examined the National Health and Nutrition Examination Survey (NHANES) database from1999 to 2014 to evaluate the effect of early natural menopause and surgical menopause on CKD prevalence as well as all-cause mortality among women with CKD respectively. The differences in the levels of sex steroid hormone between women with and without CKD were also explored.

## Methods

### Study Participants

All data in this study were from NHANES, a representative cross-section survey. It was designed to get information about civilian residents' health and nutritional status in the United States, using a multistage-stratified sample. The NHANES is a periodic survey conducted by the National Center for Health Statistics (NCHS) of the Centers for Disease Control and Prevention (CDC). The NCHS Research Ethics Review Board reviewed and approved the NHANES and all participants provided written informed consent. The NHANES Reproductive Health questionnaire surveys females to provide personal interview data on menstrual history, pregnancy history, lactation, oral contraceptive, and other related conditions. These questions were administered at the mobile examination center by trained interviewers, using the Computer-Assisted Personal Interviewing system as part of the Mobile Examination Center (MEC) interview.

**Figure 1:**
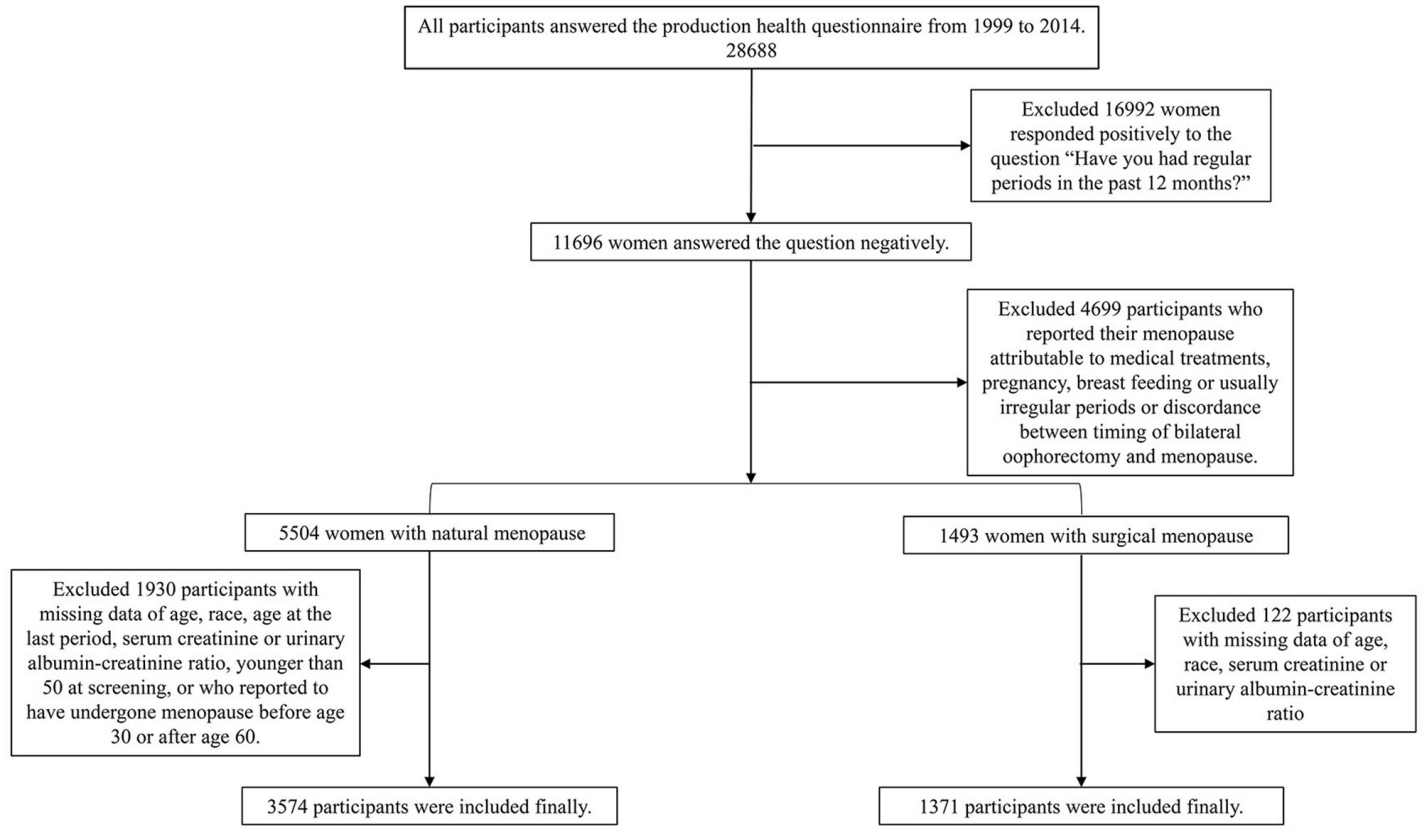
The flowchart of participants' section progress.

We merged the data from the 1999 to 2014 NHANES strictly following the analytical guidelines. 28,688 women answered the reproduction health questionnaire during this period. 10,064 post-menopause women were selected according to the negative response to the question “Have you had at least one menstrual period in the past 12 months? (Please do not include bleedings caused by medical conditions, hormone therapy, or surgeries).” We then excluded women who reported their menopause status as attributable to pregnancy, breastfeeding, usually irregular period. For the surgical menopause group, 1,515 participants reported binary oophorectomy and the surgery time is the same as age at the last period were included. For the natural menopause group, according to the questions “What is the reason that you have not had a period in the past 12 months?” “Have you had a hysterectomy, including a partial hysterectomy, that is, surgery to remove your uterus or womb?” and “Have you had at least one of ovaries removed (either when had the uterus removed or at another time)?” we excluded 2,430 women with a history of hysterectomy, unilateral oophorectomy, or women who reported menopause are due to other medical treatments. Because no data were available on age at menopause, 404 women were excluded. Since this is a questionnaire survey, considering that participants may not be clear about the question, a lot of them reported the age of menopause was the same as age at screening, and most of the participants reported extreme values to have incomplete information. To ensure the accuracy of data, we ruled out 216 women with extreme age at menopause (<30 years old or >60) and 253 women younger than 50 years old at screening consistent with the past study (15–17). Both groups exclude women with missing data of age, race, age at the last period, serum creatinine, or urinary albumin-creatinine ratio. Finally, there were 3,574 participants in the natural menopause group and 1,377 in the surgical menopause group in the present study (Figure 1).

For the lack of sex hormone data of women in 1999–2012 NHANES, these laboratory data were from Sex Steroid Hormone—Serum data file in 2013–2016 NHANES. 1,754 women were included to explore the difference in the level of sex hormones between women with CKD and without CKD in the present study.

### Definitions of Early Menopause

Natural menopause was defined as physiological amenorrhoea longer than 12 months and does not interfere with other causes, such as surgery or medical treatment (18). Surgical menopause was due to surgical removal of both ovaries before the natural age of menopause (19). Experiencing menopause before 45 was thought to be early menopause which is defined by European Menopause and Andropause Society (20, 21).

### Assessment of CKD

Data related to eGFR calculation are accessed from NHANES. Serum Cr was calibrated for the 1999–2000 and 2005–2006 participants. The recalibration equations for 1999–2000 and 2005–2006 NHANES surveys were applied: standard serum Cr (mg/dl) = 0.147 + 1.013 × uncalibrated serum Crw (mg/dl) and standard serum Cr (mg/dl) = −0.016 + 0.978 × uncalibrated serum Cr (mg/dl). Estimating glomerular filtration rate (eGFR) was calculated by the CKD Epidemiology Collaboration equation:


$$\begin{array}{l} \text{eGFR }=\;141\;\times \text{ min}\left(\text{Scr}/\text{κ},\;1\right)\text{α }\times \text{ max}\left(\text{Scr}/\text{κ},\;1\right)-1.209\\ \;\times \;0.993\text{Age }\times \;1.018\;\left[\text{if female}\right]\;/1.159\;\left[\text{if black}\right] \end{array}$$

(Scr is serum creatinine, κ is 0.7 for females and 0.9 for males, α is −0.329 for females and −0.411 for males, min indicates the minimum of Scr/κ or 1, and max indicates the maximum of Scr/κ or 1) (22). Relevant data were obtained from laboratory data of NHANES. CKD was defined as participants with an estimated glomerular filtration rate (eGFR) <60 ml/min/1.73 m^2^ or urinary albumin-to-creatinine ratio (UACR) ≥30 mg/g, and CKD patients were classified into stages according to GFR categories and albuminuria categories by the Kidney Disease: Improving Global Outcomes (KDIGO) organization (23).

### Mortality Data

Public-use Linked Mortality Files are available for continuous NHANES 1999–2014. These files provide mortality data from the date of survey enrollment through December 31, 2015. The survival time data we use are the number of Person-Months of follow-up from NHANES Interview date, with a mean follow-up time of 89.5 months. Mortality source and cause of death were determined using death certificates similar to previous studies (24, 25).

### Covariates

Clinical covariates were classified as demographics, medical conditions, and lifestyle factors. We used the information at screening from the survey in these analyses. Demographic factors included age, race/ethnicity, and marital status. Medical conditions included hypertension (defined as a history of physician-diagnosed hypertension, a measured average systolic blood pressure ≥ 140 mm Hg, diastolic blood pressure ≥ 90 mm Hg, or current use of antihypertensive medication), diabetes (defined as a history of physician-diagnosed diabetes, hemoglobin A1c level ≥ 6.5% or current use of diabetes medication). Obesity (characterized by standard body index, under 25 kg/m^2^, 25 to <30 kg/m^2^, ≧30 kg/m^2^), triglycerides (TG, mg/dl), total cholesterol (TC, mg/dl), high-density lipoprotein cholesterol (HDL-c, mg/dl), coronary heart disease, congestive heart failure, stroke, cancer were also included in this analysis. Lifestyle factors included smoking status (never, ever, current) and alcohol consumption (never, ever, current).

### Statistical Analysis

We reported the characteristics of post-menopause participants and compared those four groups, natural menopause before 45, natural menopause after 45, surgical menopause before 45, and surgical menopause after 45. For continuous variables, if data was of normality, we used ANOVA tests to compared different groups. Otherwise, non-parametric tests were used. And chi-square tests were used for classified variables. After that, we performed logistic regression to evaluate the association of early menopause with CKD prevalence and CKD stages. Model I was a crude model, while model II had been adjusted for age and race/ethnicity, and model III were further adjusted for marital status, smoke, alcohol use, obesity, TG, TC, HDL-c, hypertension, and diabetes. To explore the association between early menopause and all-cause mortality, Cox proportional hazard regression was used in all participants. CKD stage was adjusted in model I,model II was further adjusted for age and race/ethnicity. The fully adjusted model further included smoke, alcohol use, obesity, TG, TC, HDL-c, hypertension, diabetes, coronary heart disease, congestive heart failure, stroke, cancer. We also used Cox proportional hazard regression in CKD and non-CKD groups respectively. eGFR and UACR were adjusted in model I, and model II adjusted for age and race/ethnicity besides. The fully adjusted model included smoke, alcohol use, obesity, TG, TC, HDL-c, hypertension, diabetes, coronary heart disease, congestive heart failure, stroke, cancer additionally. We also performed logistic regression and Cox regression using the same process with the age of menopause as a continuous variable. Finally, we compared the differences in the levels of sex steroid hormone between people with and without CKD, using an independent-samples t-test. A 2-sided *p* < 0.05 was considered statistically significant. All data management and analyses were performed in SPSS version 26.0.

## Results

### Description of the Study Population

Table 1 described the characteristics of the women included in the study. Mean ± SD age was 64 ± 10.2 years of all participants, while the mean age at menopause was 47 ± 7.1 years. Women with early menopause, both natural and surgical, had a higher percentage of current smoking and having cardiovascular disease including stroke and congestive heart failure. In addition, the early menopause groups had a higher proportion of having CKD. Compared with people with natural menopause, women who had undergone surgical menopause tended to be obese, have cancer and hypertension, and have a higher level of TC. There were also significant differences in race, marital status, and alcohol use among the groups. The CKD stages of each group were also listed (Supplementary Table 1), few CKD women had UACR <3 mg/mmol, and most of them had a decrease in eGFR.

**Table 1:** Characteristics of the participants.

**Characteristics**	**Natural menopause at age ≧45 y (*n* = 3,038)**	**Natural menopause at age <45 y (*n* = 536)**	**Surgical menopause at age ≧45 y (*n* = 516)**	**Surgical menopause at age <45 y (*n* = 815)**	***P* value**
Age, y	64.0 (58.0–72.0)	65.0 (58.0–74.0)	65.0 (59.0–73.0)	62.0 (52.0–71.0)	<0.001
Race/ethnicity, No. (%)					<0.001
Non-hispanic white	1,603 (52.8)	258 (48.1)	307 (59.5)	488 (59.9)	
Non-hispanic black	492 (16.2)	90 (16.8)	98 (19.0)	169 (20.7)	
Other	943 (31.0)	188 (35.1)	111 (21.5)	158 (19.4)	
Marital status, No. (%)					<0.001
Married/ cohabitating	1,544 (50.8)	226 (42.2)	296 (57.4)	434 (53.2)	
Widowed/seperated/divorced	1,311 (43.2)	269 (50.2)	199 (38.6)	339 (41.6)	
Never married	183 (6.0)	41 (7.6)	21 (4.1)	42 (5.2)	
Alcohol use, No. (%)					0.001
Never	751 (24.7)	158 (29.5)	98 (19.0)	182 (22.3)	
Ever	642 (21.1)	115 (21.5)	134 (26.0)	198 (24.3)	
Current	1,645 (54.2)	263 (49.1)	284 (55.0)	435 (53.4)	
Smoke, No. (%)					<0.001
Never	1,879 (61.9)	307 (57.3)	320 (62.0)	431 (52.8)	
Ever	820 (27.0)	135 (25.2)	151 (29.3)	223 (27.4)	
Current	339 (11.2)	94 (17.5)	45 (8.7)	161 (19.8)	
Hypertension, No. (%)	1,836 (60.4)	341 (63.6)	356 (69.0)	520 (63.8)	0.001
Diabetes, No. (%)	621 (20.4)	123 (23.0)	106 (20.5)	175 (21.5)	0.583
Body mass index (kg/m^2^), No. (%)					0.023
<25	885 (29.1)	140 (26.1)	130 (25.2)	191 (23.4)	
25– <30	946 (31.1)	177 (33.0)	164 (31.8)	257 (31.5)	
≧30	1,207 (39.7)	219 (40.9)	222 (43.0)	367 (45.0)	
Triglycerides (mg/dl)	125.5 (88.0–184.0)	126.0 (86.3–179.3)	136.0 (93.0–194.0)	132.0 (92.0–198.0)	0.008
Total cholesterol (mg/dl)	209.0 (183.0–237.0)	207.0 (177.3–233.0)	210.5 (181.0–236.0)	207.0 (181.0–237.0)	0.215
HDL-cholesterol (mg/dl)	57.0 (47.0–68.0)	56.0 (46.0–68.0)	57.0 (48.0–69.8)	56.0 (46.0–68.0)	0.360
Congestive heart failure, No. (%)	109 (3.6)	36 (6.7)	23 (4.5)	45 (5.5)	0.003
Coronary heart disease, No. (%)	127 (4.2)	31 (5.8)	25 (4.8)	41 (5.0)	0.340
Stroke, No. (%)	133 (4.4)	32 (6.0)	24 (4.7)	68 (8.3)	<0.001
Cancer, No. (%)	368 (12.1)	65 (12.1)	104 (20.2)	160 (19.6)	<0.001
Ovary cancer, No. (%)	1 (0.0)	0 (0.0)	3 (0.0)	15 (0.0)	/
eGFR, ml/min/1.73 m^2^	81.7 (66.8–94.7)	78.8 (62.4–93.2)	80.9 (66.4–95.9)	82.2 (66.4–100.5)	<0.001
UACR, mg/g	9.1 (5.8–18.4)	9.8 (6.3–23.5)	8.1 (5.4–15.8)	8.4 (5.4–18.3)	<0.001
CKD, No. (%)	829 (27.3)	180 (33.3)	140 (27.1)	237 (29.1)	0.023
Death, No. (%)	485 (16.0)	116 (21.6)	73 (14.1)	129 (15.8)	0.004

### Early Menopause and CKD Prevalence

We used logistics regressions to compare the CKD risk between the early menopause group and the population experiencing menopause after 45 (Table 2). For the natural menopause group, in crude analysis, women with early natural menopause were more likely to have CKD [OR = 1.35 (1.11–1.64), *p* = 0.003], the model adjusted for demographics factors and the fully adjusted model both supported the result that women with early natural menopause tend to have CKD, and it was significant. For the surgical menopause group, although the unadjusted model showed there was no correlation between early surgical menopause and CKD, women with early surgical menopause had a significant risk of CKD after adjusted for age and race [OR = 1.41 (1.08–1.83), *p* = 0.011], the result remained the same as the further adjusted model. However, when we explored the relationship between early menopause and CKD-G1/2 and CKD-G3~5 respectively (Supplementary Tables 2, 3), except early natural menopause was associated with CKD-G3~5 [OR = 1.37 (1.04–1.80), *p* = 0.025], there were no other significant findings. The regression which treated age at menopause as a continuous variable and included surgical vs. non-surgical as a covariate showed the same results (Supplementary Table 4). Older menopausal age was associated with a lower risk of CKD [OR = 0.98 (0.97–0.99), *p* = 0.002].

**Table 2:** The association of early menopause and CKD prevalence by logistic regression.

**Groups**	**Model I^a^ OR**	***P* value**	**Model II^b^ OR**	***P* value**	**Model III^c^ OR**	***P* value**	***P* value of Hosmer/Lemeshow**
**Women with natural menopause**							
Natural menopause at age ≧45	Ref (1.00)		Ref (1.00)		Ref (1.00)		
Early natural menopause	1.35 (1.11–1.64)	0.003	1.26 (1.02–1.55)	0.034	1.25 (1.00–1.55)	0.046	0.209
**Women with surgical menopause**							
Surgical menopause at age ≧45	Ref (1.00)		Ref (1.00)		Ref (1.00)		
Early surgical menopause	1.10 (0.86–1.41)	0.442	1.41 (1.08–1.83)	0.011	1.33 (1.01–1.76)	0.041	0.554

a *Unadjusted model*.

b *Adjusted for age and race/ethnicity*.

c *Adjusted for age, race/ethnicity, marital status, smoke, alcohol use, obesity, triglycerides (mg/dl), total cholesterol (mg/dl), HDL-cholesterol (mg/dl), hypertension, and diabetes*.

### Early Menopause and All-Cause Mortality

When we treated the CKD stage as a covariate, both early natural menopause and surgical menopause were risk factors for survival (Table 3). Then we separated all participants into two groups according to conditions of CKD and non-CKD. For the CKD group, either early natural or surgical menopause had no significant correlation with all-cause mortality (Table 4). For the non-CKD group (Table 5), although the result of natural menopause is similar to the CKD group, surgical menopause differed from the CKD group. Higher mortality risk was observed in the non-CKD population with early surgical menopause [HR = 1.62 (1.06–2.49), *p* = 0.027]. However, when we treated age at menopause as a continuous variable (Supplementary Table 5), we found early natural menopause in the CKD group was associated with higher mortality risk [HR = 0.98 (0.96–1.00), *p* = 0.024]. The other results made no difference.

**Table 3:** Association of early menopause and all-cause mortality by Cox proportional hazard regression models.

**Groups**	**Model l^a^ HR**	***P* value**	**C-index**	**Model II^b^ HR**	***P* value**	**C-index**	**Model III^c^ HR**	***P* value**	**C-index**
**Women with natural menopause**									
Natural menopause at age ≧45	Ref (1.00)			Ref (1.00)			Ref (1.00)		
Early natural menopause	1.26 (1.03–1.55)	0.025	0.75 (0.72–0.77)	1.19 (0.97–1.45)	0.101	0.76 (0.74–0.78)	1.23 (1.00–1.51)	0.048	0.79 (0.78–0.80)
**Women with surgical menopause**									
Surgical menopause at age ≧45	Ref (1.00)			Ref (1.00)			Ref (1.00)		
Early surgical menopause	1.14 (0.85–1.52)	0.379	0.73 (0.69–0.78)	1.51 (1.13–2.01)	0.005	0.78 (0.75–0.82)	1.46 (1.09–1.97)	0.012	0.81 (0.78–0.84)

a *Adjusted for CKD stage*.

b *Adjusted for age, race/ethnicity, and CKD stage*.

c *Adjusted for age, race/ethnicity, CKD stage, marital status, smoke, alcohol use, obesity, triglycerides (mg/dl), total cholesterol (mg/dl), HDL-cholesterol (mg/dl), hypertension, diabetes, coronary heart disease, congestive heart failure, stroke, cancer*.

**Table 4:** Association of early menopause and all-cause mortality by Cox proportional hazard regression models among CKD group.

**Groups**	**Model l^a^ HR**	***P* value**	**C-index**	**Model II^b^ HR**	***P* value**	**C-index**	**Model III^c^ HR**	***P* value**	**C-index**
**Women with natural menopause**									
Natural menopause at age ≧45	Ref (1.00)			Ref (1.00)			Ref (1.00)		
Early natural menopause	1.25 (0.96–1.63)	0.095	0.64 (0.58–0.65)	1.20 (0.92–1.57)	0.177	0.69 (0.66–0.72)	1.22 (0.93–1.60)	0.158	0.73 (0.70–0.76)
**Women with surgical menopause**									
Surgical menopause at age ≧45	Ref (1.00)			Ref (1.00)			Ref (1.00)		
Early surgical menopause	1.10 (0.74–1.63)	0.640	0.62 (0.56–0.68)	1.40 (0.94–2.08)	0.096	0.73 (0.68–0.78)	1.25 (0.81–1.92)	0.317	0.76 (0.71–0.81)

a *Adjusted for eGFR and UACR*.

b *Adjusted for age, race/ethnicity, eGFR, and UACR*.

c *Adjusted for age, race/ethnicity, eGFR, UACR, marital status, smoke, alcohol use, obesity, triglycerides (mg/dl), total cholesterol (mg/dl), HDL-cholesterol (mg/dl), hypertension, diabetes, coronary heart disease, congestive heart failure, stroke, cancer*.

**Table 5:** Association of early menopause and all-cause mortality by Cox proportional hazard regression models among the non-CKD group.

**Groups**	**Model I^a^ HR**	***P* value**	**C-index**	**Model II^b^ HR**	***P* value**	**C-index**	**Model III^c^ HR**	***P* value**	**C-index**
**Women with natural menopause**									
Natural menopause at age ≧45	Ref (1.00)			Ref (1.00)			Ref (1.00)		
Early natural menopause	1.28 (0.93–1.76)	0.123	0.65 (0.61–0.69)	1.22 (0.89–1.68)	0.226	0.72 (0.69–0.76)	1.12 (0.801–1.55)	0.495	0.74 (0.71–0.78)
**Women with surgical menopause**									
Surgical menopause at age ≧45	Ref (1.00)			Ref (1.00)			Ref (1.00)		
Early surgical menopause	1.44 (0.95–2.17)	0.087	0.61 (0.54–0.69)	1.67 (1.10–2.53)	0.015	0.73 (0.67–0.79)	1.62 (1.06–2.49)	0.027	0.78 (0.73–0.83)

a *Adjusted for eGFR and UACR*.

b *Adjusted for age, race/ethnicity, eGFR, and UACR*.

c *Adjusted for age, race/ethnicity, eGFR, UACR, marital status, smoke, alcohol use, obesity, triglycerides (mg/dl), total cholesterol (mg/dl), HDL-cholesterol (mg/dl), hypertension, diabetes, coronary heart disease, congestive heart failure, stroke, cancer*.

### The Hormone Differences Between CKD and Non-CKD Group

To find out the connection between the CKD prevalence and early menopause in women, we compared the hormone level of CKD and the non-CKD group (Table 6). Several hormones showed a significant difference. CKD patients had a higher level of follicle-stimulating hormone (34.44 vs. 27.71, *p* = 0.029) and luteinizing hormone (22.66 vs. 19.63, *p* = 0.029), but a lower level of testosterone (153.37 vs. 176.29, *p* = 0.033) and estradiol (29.50 vs. 40.11, *p* = 0.001). For sex hormone-binding globulin (SHBG), CKD and non-CKD groups made no difference.

**Table 6:** The difference in hormones between the population with and without CKD.

**Variable**	**CKD group (*n* = 201)**	**Non-CKD group (*n* = 1,553)**	***P* value**
Follicle stimulating hormone (mIU/ml)	34.44	27.71	0.029
luteinizing hormone (mIU/ml)	22.66	19.63	0.029
Testosterone, total (ng/dl)	153.37	176.29	0.033
Estradiol (pg/ml)	29.50	40.11	0.001
SHBG (nmol/l)	61.43	57.48	0.782

## Discussion

In the present study, both early natural menopause and early surgical menopause were associated with statistically significant increased risk of CKD, with persistent relation observed after adjusted for conventional factors. In our study, when we treated the CKD stage as a covariate, both early natural menopause and surgical menopause were risk factors for survival, but the results had some differences when we divided the participants into CKD and non-CKD. Early natural menopause was independent of longevity in the non-CKD group. However, in the CKD group, the regression results of the age of menopause as a continuous variable and early menopause as a categorical variable are different. As for surgical menopause, bilateral oophorectomy before age 45 years increased the all-cause mortality in the non-CKD group, but such finding was not observed in the CKD group. Our study also revealed the apparent differences in sex hormone levels between CKD and non-CKD participants.

Our study revealed women with early menopause, both natural and surgical, had a higher percentage of current smoking and having cardiovascular disease including stroke and congestive heart failure. Studies have reported that smoking is associated with an earlier age of menopause, and a later age of menopause could reduce the risk of cardiovascular diseases (26). Our study confirmed this. In our study, compared with people with natural menopause, women who had undergone surgical menopause tended to be obese, have cancer and hypertension, and have a higher level of TC. This indicates that cancer may be an important cause of oophorectomy. Obesity, hyperlipidemia, and hypertension are associated with a higher risk of ovarian diseases (27), which make women more likely to undergo oophorectomy. There were also significant differences in race, marital status, and alcohol use among the groups. A meta-analysis has shown low and moderate alcohol consumption was associated with later menopause onset, compared to non-drinkers (28). In addition, alcohol may also cause lesions in the female reproductive organs leading to oophorectomy (29).

Our study suggested that early menopause, both natural and surgical, was associated with a higher risk of CKD and early natural menopause is mainly associated with CKD-G3~5. A study based on the multiethnic Women's Health Initiative cohort had mentioned early natural menopause occurred more among women with CKD (3), which indicated a potential association between early natural menopause and CKD. Another cohort study found women who had undergone bilateral oophorectomy were more likely to develop CKD compared to age-matched referent women without surgery (30), but the relationship between the age at surgery and CKD prevalence is unclear. In this study, women who had bilateral oophorectomy before age 45 were at a higher risk of CKD than those who had surgery after age 45, suggesting the association between early surgical menopause and CKD prevalence.

The difference in sex hormone levels between the CKD group and the non-CKD group in this study proved that sex hormones may play crucial roles in the association between early menopause and CKD. As CKD develops, an inadequate cyclic release of gonadotropin-releasing hormone (GnRH) by the hypothalamus leads to loss of normal pulsatile gonadotropin secretion by the pituitary including FSH and LH (31–34). In this case, the pre-ovulatory rise of estrogen and progesterone is absent, causing ovulatory obstacles, then amenorrhea occurs, finally resulting in early menopause (35). On the other hand, after menopause, the level of estrogen declines, which may accelerate the progression of glomerulosclerosis (36). A recent study had mentioned that the risk for CKD was higher in women with shorter reproductive life span duration, indicating less endogenous estrogen exposure was closely associated with higher CKD risk in postmenopausal women (37). In addition, in patients with surgical menopause, surgery and subsequent drug use might increase the renal load and lead to injury of renal. Therefore, the relationship between early menopause and CKD is bi-directional. Nevertheless, the mechanism that confers the association of early menopause and CKD is uncertain.

In our study, early natural menopause did no effect on mortality in the non-CKD group. In the CKD group, there was a negative result when we take early menopause as a categories variable, however, when we treated age at menopause as a continuous variable, we found early natural menopause was associated with higher mortality. The relation between age at natural menopause and mortality is controversial. A study based on the Health, Well-Being and Aging study found women with early menopause had a 48% higher risk of all-cause mortality compared to the women with menopause at normal age (38), but a Taiwan study showed there was no significant difference in all-cause mortality between women with early menopause and the normal reference (39). Early menopause is associated with many other diseases and might result in an uncertain relationship between early menopause and mortality. Previous research has shown that early onset of menopause was connected with a higher risk of cardiovascular disease mortality (12, 40), whereas early menopause was known to decrease the risk of developing breast cancer, ovarian and endometrial cancer (41–43). These factors may also affect the longevity of postmenopausal women. More studies are needed to confirm the association of early natural menopause with mortality.

Bilateral oophorectomy undertaken before age 45 years is thought to increase mortality in the general population (44, 45), the similar conclusion was observed in the non-CKD group in our study. Oophorectomy-induced estrogen deficiency may partly explain the surgery's damage to survival. A low level of estrogen increases the risk of cardiovascular disease, osteoporosis, or neurological disease. Consequently, it influences the longevity of these patients (11). There was no data in the NHANES on why women had bilateral oophorectomy, but other studies have reported although some oophorectomies are performed to treat ovary-related pathology or to prevent ovarian cancer in women at increased risk of ovarian cancer, most oophorectomies are done in cases of grossly normal ovaries in women at average risk for ovarian cancer (46). Women who underwent hysterectomy and had mutations in the BCRA2 and BRCA1 genes were most likely to have prophylactic bilateral oophorectomy (47). Hysterectomy and gene mutations may also be the risk factors of mortality. Moreover, early menopause has been linked to psychological disorders such as depression and anxiety, which can accelerate the progression of diseases and increase mortality (48). However, in the CKD population we studied, early bilateral oophorectomy was irrelevant with survival. There were several possible explanations for this result. CKD may interfere with the pathogenesis of menopause. For example, Vasomotor symptoms occur in 30–80% of post-menopausal women in the form of peripheral vasodilation and sweating, while women with CKD have a lower risk to experience it (3). In addition, decreased estradiol catabolism in CKD patients might lead to a higher free estradiol level (49). Moreover, taking the same dose of estradiol, serum concentrations of estradiol and estrone in patients with CKD are 2–3 times higher than in healthy postmenopausal women (50) and improved the treatment efficacy of estradiol supplement after the bilateral oophorectomy. Besides, for the regular medical examinations during the perioperative period of oophorectomy, CKD patients could be early diagnosed, which is also conducive to survival. The above factors may counteract the adverse effects of surgical menopause. However, the exact mechanisms remain inconclusive.

There are still many limitations in our study. First, the criterion of menopause was only 1 year without menstruation after excluding some special conditions, without considering the issue of hormone levels. Second, most of the data were from the questionnaire, including the time of menopause. Although some abnormal data had been screened out, the accuracy of the data was still lacking. As for women with surgical menopause, there are no specific reasons for women to undergo bilateral oophorectomy in the NHANES, and we can only speculate it according to other studies, that might interfere with the outcome. Additionally, there were inevitable censorings in the follow-up, which may have an influence on the results. Finally, because we can't get information about when the participants developed CKD, the sequence of CKD and menopause is unknown and the causal relationship between early menopause and CKD is not clear and requires further cohort studies. Despite these limitations, our study has the merit of using a representative national sample, and we have found evidence in support of the association between early menopause and CKD prevalence and all-cause mortality, and their difference between CKD and non-CKD patients.

## Conclusion

Both early natural and surgical menopause were associated with a higher risk of CKD. In our study, early surgical menopause was a hazard factor for survival in the non-CKD group, but not in the CKD group. The mechanisms behind these associations need further research.

## Data Availability Statement

The original contributions presented in the study are included in the article/Supplementary Material, further inquiries can be directed to the corresponding author/s.

## Ethics Statement

The NHANES is a periodic survey conducted by the National Center for Health Statistics (NCHS) of the Centers for Disease Control and Prevention (CDC). The NCHS Research Ethics Review Board reviewed and approved the NHANES and all participants provided written informed consent. NCHS Ethics Review Board Protocol Number: Protocol #98-12, Protocol #2005-06 and Protocol #2011-17.

## Author Contributions

GX: conceptualization. DQ and Z-fW: data curation and writing—original draft. Z-fW: formal analysis. S-WG: methodology and writing—review and editing. DQ: resources and software. Y-cC and RL: validation. All authors contributed to the article and approved the submitted version.

## Funding

This work was financially supported by the International (Regional) Cooperation and Exchange Projects (NSFC-DFG Grant No. 81761138041), National Natural Science Foundation of China (Grant No. 81570667), Major Research Plan of the National Natural Science Foundation of China (Grant No. 91742204), and the National Key R&D Program of China (Grant No. 2018YFC1314003-1).

## Conflict of Interest

The authors declare that the research was conducted in the absence of any commercial or financial relationships that could be construed as a potential conflict of interest.

## Publisher's Note

All claims expressed in this article are solely those of the authors and do not necessarily represent those of their affiliated organizations, or those of the publisher, the editors and the reviewers. Any product that may be evaluated in this article, or claim that may be made by its manufacturer, is not guaranteed or endorsed by the publisher.
