# Supplementary material for: Early Menopause May Associate With a Higher Risk of CKD and All-Cause Mortality in Postmenopausal Women: An Analysis of NHANES, 1999–2014
Source: Front Med (Lausanne). 2022 Mar 18;9:823835. doi: 10.3389/fmed.2022.823835 (PMC8971204; doi:10.3389/fmed.2022.823835)
Supplement: Supplementary file 1 [file Data_Sheet_1.docx]

Supplementary Material

**Supplementary Figures and Tables**

**Supplementary Table S1: CKD stages of the participants with CKD**

|  | **Natural menopause at age≧45y(n=829)** | **Natural menopause at age <45y(n=180)** | **Surgical menopause at age ≧45y(n=140)** | **Surgical menopause at age <45y(n=237)** | **P value** |
| --- | --- | --- | --- | --- | --- |
| CKD stages |  |  |  |  | 0.005 |
| G1A2 | 0(0.0) | 0(0.0) | 0(0.0) | 1(0.0) |  |
| G1A3 | 131(15.8) | 18(10.0) | 25(17.9) | 43(18.1) |  |
| G2A2 | 1(0.0) | 0(0.0) | 1(0.0) | 1(0.0) |  |
| G2A3 | 198(23.9) | 47(26.1) | 22(15.7) | 47(19.8) |  |
| G3aA1 | 14(0.0) | 2(1.1) | 4(2.9) | 8(3.4) |  |
| G3aA2 | 272(32.8) | 47(26.1) | 36(25.7) | 67(28.3) |  |
| G3aA3 | 65(7.8) | 21(11.7) | 10(7.1) | 16(6.7) |  |
| G3bA1 | 4(0.0) | 0(0.0) | 4(2.9) | 1(0.0) |  |
| G3bA2 | 57(6.1) | 23(12.8) | 22(15.7) | 27(11.4) |  |
| G3bA3 | 45(5.4) | 9(5.0) | 8(5.7) | 13(5.5) |  |
| G4A1 | 0(0.0) | 1(0.0) | 0(0.0) | 0(0.0) |  |
| G4A2 | 15(1.8) | 4(2.2) | 1(0.0) | 4(1.7) |  |
| G4A3 | 17(2.1) | 6(3.3) | 5(3.6) | 7(3.0) |  |
| G5A1 | 0(0.0) | 0(0.0) | 0(0.0) | 0(0.0) |  |
| G5A2 | 1(0.0) | 0(0.0) | 0(0.0) | 1(0.0) |  |
| G5A3 | 9(1.0) | 2(1.1) | 2(1.4) | 1(0.0) |  |

**Supplementary Table S2: The association of early menopause and CKD-G1/2 prevalence by logistic regression.**

| **Groups** | **Model Ⅰ^a^ OR** | **P value** | **Model Ⅱ^b^ OR** | **P value** | **Model Ⅲ^c^ OR** | **P value** | **P value of Hosmer/Lemeshow** |
| --- | --- | --- | --- | --- | --- | --- | --- |
| Women with natural menopause |  |  |  |  |  |  |  |
| Natural menopause at age ≧45 | Ref(1.00) |  | Ref(1.00) |  | Ref(1.00) |  |  |
| Early natural menopause | 1.22(0.92-1.63) | 0.173 | 1.17(0.87-1.56) | 0.306 | 1.16(0.86-1.56) | 0.344 | 0.726 |
|  |  |  |  |  |  |  |  |
| Women with surgical menopause |  |  |  |  |  |  |  |
| Surgical menopause at age ≧45 | Ref(1.00) |  | Ref(1.00) |  | Ref(1.00) |  |  |
| Early surgical menopause | 1.25(0.86-1.81） | 0.246 | 1.42(0.97-2.07） | 0.073 | 1.36(0.92-2.02) | 0.124 | 0.222 |

1. unadjusted model.
2. adjusted for age and race/ethnicity.
3. adjusted for age, race/ethnicity, marital status, smoke, alcohol use, obesity, triglycerides (mg/dL), total cholesterol (mg/dL), HDL-cholesterol (mg/dL), hypertension, diabetes and cancer.

**Supplementary Table S3: The association of early menopause and CKD-G3~5 prevalence by logistic regression.**

| **Groups** | **Model Ⅰ^a^ OR** | **P value** | **Model Ⅱ^b^ OR** | **P value** | **Model Ⅲ^c^ OR** | **P value** | **P value of Hosmer/Lemeshow** |
| --- | --- | --- | --- | --- | --- | --- | --- |
| Women with natural menopause |  |  |  |  |  |  |  |
| Natural menopause at age ≧45 | Ref(1.00) |  | Ref(1.00) |  | Ref(1.00) |  |  |
| Early natural menopause | 1.43(1.13-1.80） | 0.002 | 1.37(1.05-1.79） | 0.021 | 1.37(1.04-1.80) | 0.025 | 0.672 |
|  |  |  |  |  |  |  |  |
| Women with surgical menopause |  |  |  |  |  |  |  |
| Surgical menopause at age ≧45 | Ref(1.00) |  | Ref(1.00) |  | Ref(1.00) |  |  |
| Early surgical menopause | 1.03(0.77-1.37） | 0.867 | 1.30(0.94-1.80） | 0.109 | 1.26(0.90-1.77) | 0.180 | 0.115 |

1. unadjusted model.
2. adjusted for age and race/ethnicity.
3. adjusted for age, race/ethnicity, marital status, smoke, alcohol use, obesity, triglycerides (mg/dL), total cholesterol (mg/dL), HDL-cholesterol (mg/dL), hypertension, diabetes and cancer.

**Supplementary Table S4: Association between age at menopause and CKD prevalence by logistic regression.**

|  | **Age at menopause** | **P value** | **P value of Hosmer/Lemeshow** |
| --- | --- | --- | --- |
| **Model Ⅰ** | 0.99(0.98-1.00) | 0.048 | 0.014 |
| **Model Ⅱ** | 0.98(0.97-0.99) | <0.001 | <0.001 |
| **Model Ⅲ** | 0.98(0.97-0.99) | 0.002 | 0.143 |

1. adjusted for menopause type.
2. adjusted for age, race/ethnicity and menopause type.
3. adjusted for age, race/ethnicity, marital status, smoke, alcohol use, obesity, triglycerides (mg/dL), total cholesterol (mg/dL), HDL-cholesterol (mg/dL), hypertension, diabetes.

**Supplementary Table S5: Association of age at menopause and all-cause mortality by Cox proportional hazard regression models among CKD group**

| **Groups** | **Model l ^a^ HR** | **P value** | **Model Ⅱ^b^ HR** | **P value** | **Model Ⅲ^c^ HR** | **P value** |
| --- | --- | --- | --- | --- | --- | --- |
| CKD women with natural menopause | 0.98(0.96-1.00) | 0.021 | 0.98(0.96-1.00) | 0.031 | 0.98(0.96-1.00) | 0.024 |
| CKD women with surgical menopause | 0.99(0.97-1.01) | 0.540 | 0.98(0.95-1.00) | 0.031 | 0.98(0.95-1.00) | 0.061 |
|  |  |  |  |  |  |  |
| Non-CKD women with natural menopause | 0.98(0.95-1.00) | 0.059 | 0.98(0.95-1.00) | 0.076 | 0.99(0.96-1.01) | 0.198 |
| Non-CKD women with surgical menopause | 0.97(0.95-1.00) | 0.041 | 0.97(0.94-0.99) | 0.006 | 0.97(0.94-0.99) | 0.011 |

1. adjusted for eGFR and UACR.
2. adjusted for age, race/ethnicity, eGFR, and UACR.
3. adjusted for age, race/ethnicity, eGFR, UACR, marital status, smoke, alcohol use, obesity, triglycerides (mg/dL), total cholesterol (mg/dL), HDL-cholesterol (mg/dL), hypertension, diabetes, coronary heart disease, congestive heart failure, stroke, cancer
